# MSC therapy ameliorates experimental gouty arthritis hinting an early COX-2 induction

**DOI:** 10.3389/fimmu.2023.1193179

**Published:** 2023-07-18

**Authors:** Juan Pablo Medina, Ismael Bermejo-Álvarez, Sandra Pérez-Baos, Rosa Yáñez, María Fernández-García, Damián García-Olmo, Aránzazu Mediero, Gabriel Herrero-Beaumont, Raquel Largo

**Affiliations:** ^1^ Bone and Joint Research Unit, Rheumatology Dept, IIS-Fundación Jiménez Díaz Universidad Autonoma de Madrid (UAM), Madrid, Spain; ^2^ Hematopoietic Innovative Therapies Division, Centro de Investigaciones Energéticas, Medioambientales y Tecnológicas (CIEMAT) and Centro de Investigación Biomédica en Red de Enfermedades Raras (CIBER-ER), Madrid, Spain; ^3^ Advanced Therapies Dept, IIS-Fundación Jiménez Díaz UAM, Madrid, Spain; ^4^ New Therapies Laboratory, IIS-Fundación Jiménez Díaz UAM, Madrid, Spain; ^5^ Department of Surgery, Fundación Jiménez Díaz University Hospital, Madrid, Spain; ^6^ Department of Surgery, School of Medicine UAM, Madrid, Spain

**Keywords:** mesenchymal stem cells (MSCs), innate inflammation, macrophage, polarization, inflammasome, prostaglandin E2, COX-2

## Abstract

**Objective:**

The specific effect of Adipose-Derived Mesenchymal Stem Cells (Ad-MSC) on acute joint inflammation, where the response mostly depends on innate immunity activation, remains elusive. The pathogenesis of gouty arthritis, characterized by the deposition of monosodium urate (MSU) crystals in the joints, associated to acute flares, has been associated to NLRP3 inflammasome activation and subsequent amplification of the inflammatory response. Our aim was to study the effect of human Ad-MSC administration in the clinical inflammatory response of rabbits after MSU injection, and the molecular mechanisms involved.

**Methods:**

Ad-MSC were administered by intraarterial route shortly after intraarticular MSU crystal injections. Joint and systemic inflammation was sequentially studied, and the mechanisms involved in NLRP3 inflammasome activation, and the synthesis of inflammatory mediators were assessed in the synovial membranes 72h after insult. Ad-MSC and THP-1-derived macrophages stimulated with MSU were co-cultured in transwell system.

**Results:**

A single systemic dose of Ad-MSC accelerated the resolution of local and systemic inflammatory response. In the synovial membrane, Ad-MSC promoted alternatively M2 macrophage presence, inhibiting NLRP3 inflammasome and inducing the production of anti-inflammatory cytokines, such as IL-10 or TGF-β, and decreasing nuclear factor-κB activity. Ad-MSC induced a net anti-inflammatory balance in MSU-stimulated THP-1 cells, with a higher increase in IL-10 and IDO expression than that observed for IL-1β and TNF.

**Conclusion:**

Our *in vivo* and *in vitro* results showed that a single systemic dose of Ad-MSC decrease the intensity and duration of the inflammatory response by an early local COX-2 upregulation and PGE_2_ release. Ad-MSCs suppressed NF-kB activity, NLRP3 inflammasome, and promoted the presence of M2 alternative macrophages in the synovium. Therefore, this therapeutic approach could be considered as a pharmacological alternative in patients with comorbidities that preclude conventional treatment.

## Introduction

First studies employing MSCs as cell therapy arose from their regenerative potential, with the hypothesis that they could replace damaged cells in pathologies characterized by tissue destruction (1). However, further studies suggested that the improvement of tissue injuries could be based on the ability of MSCs to regulate both the adaptive and innate immune response, through the release of different mediators or by cell-cell contact (2, 3). However, cell therapy has achieved little success in translating promising results from pre-clinical models into clinical practice in various diseases, including chronic, immune and inflammatory disorders (4). Possible reasons for this failure could be that the experimental models might not faithfully reproduce human diseases, or that the beneficial effects of MSCs are a consequence of the inhibition of pathways that are not well-defined in these models. To date, most studies have been conducted in chronic diseases (5), where both the adaptive and innate immune responses are integrated, taking place at the same time induction and resolution processes associated with the chronic condition. This complex network of interactions makes it difficult to dissect, in an *in vivo* scenario, the mechanisms purely associated to the activation of innate immunity and its regulation by MSCs. Undoubtedly, to describe how MSCs act in the modulation of the innate immune response, we must resort to models of acute self-limited inflammation, in which these mechanisms can be adequately assessed. Acute gout is a prototype of joint disease mediated by the innate immune response (6). It is characterized by recurrent flares of articular and periarticular swelling, redness, and stiffness, together with pain. It usually resolves spontaneously within a few days leaving minimal residual lesions, even without intervention. Gout is caused by the deposition of monosodium urate (MSU) crystals into the joints, which induces a massive infiltration of neutrophils and monocytes. This early inflammatory phase is characterized by IL-1β production, which induces the release of different chemokines, cytokines, adhesion molecules which, in turn, increments cell infiltration. The release of IL-1β is driven by NOD-like receptor family pyrin domain containing-3 (NRLP3) inflammasome activation induced by MSU crystals in myeloid cells (7, 8). Upon sensing a priming signal, there is a robust increase in the gene transcription of NLRP3, pro-IL-1β, and pro-IL-18 *via* nuclear factor (NF)-κB activation, thus providing an abundance of protein for downstream processing. A second signal sensed by NLRP3 unchains the assembly of the inflammasome complex (e.g. NLRP3, Apoptosis-associated Speck-like protein (ASC) and Caspase-1), which subsequently triggers the cleavage of pro-interleukin- 1β and 18 and the release of the active cytokines. NLRP3 inflammasome is present in monocyte cell lineage responsible for the innate immune response in metabolic, autoimmune and auto-inflammatory diseases, and therefore targeting its activity could be an effective approach of reducing inflammatory response (9, 10). Our previous work points out that MSU-induced arthritis in rabbit knees show a pronounced self-limited inflammation in the joint, being more than a suitable animal model to reproduce an acute arthritic flare and to study inflamed synovial membranes (11).

MSCs actively interact with various types of innate immune cells, such as dendritic cells, natural killers, neutrophils and macrophages (12). For instance, MSCs activated by inflamed tissue macrophages release different mediators, such as prostaglandin E2 (PGE_2_) (13), TNF-stimulated gene (TSG)-6 or indoleamine 2,3-dioxygenase (IDO) (14, 15), inhibiting macrophage activity, inducing the M2 anti-inflammatory polarization, or attenuating NF-κB activation (14, 16, 17). Recent *in vitro* and *in vivo* data have revealed that MSCs inhibit NLRP3 inflammasome (18, 19). However, the anti-inflammatory effect of MSCs on acute joint inflammation has never been described before. MSCs could therefore be a promising therapeutic approach for the treatment of acute gout flare-ups, providing an excellent model to study the anti-inflammatory effect of MSCs and their mechanisms of action on innate immunity.

The main goal of this work was to study the effect of human Adipose-derived-MSCs (Ad-MSC) in an *in vivo* model of a self-limited acute gouty arthritis in rabbits. Furthermore, we aim to study the effect of Ad-MSC administration on NLRP3 activation, macrophage polarization and inflammatory activity both *in vivo* and *in vitro*.

## Materials and methods

### MSCs generation and culture

Ad-MSC were isolated from healthy donor lipoaspirates after informed consent according to institutional guidelines and the approval by the Ethics Committee of Hospital Fundación Jiménez Díaz. Ad-MSC were obtained as previously described (20). In brief, adipose tissue was disaggregated and digested with 2 mg/ml collagenase A (Serva, Heidelberg, Germany). Cells obtained after filtration and centrifugation were cultured in Minimum Essential Medium α (α-MEM; Gibco/Life Technologies/Thermo Fisher Scientific, Waltham, USA) supplemented with 5% platelet lysate (Cook Medical, IN, USA), 1% penicillin/streptomycin (Gibco/Life Technologies/Thermo Fisher Scientific, Waltham, USA), and 1 ng/ml human basic fibroblast growth factor (bFGF; Peprotech, NJ, USA). For the expansion of Ad-MSC, adherent cells were seeded at a density of 3x10^3^ cells/cm^2^ and the cell medium was changed every 2–4 days. Cells were serially passaged using 0.25% trypsin/EDTA (Sigma Aldrich, St. Louis, MO, USA) upon reaching near confluence (70%–90%). Cultured Ad-MSC showed a fibroblast-like morphology and their immunophenotype analysis confirmed that they fulfilled all the International Society of Cell Therapy criteria as described (20, 21). Ad-MSC differentiation capacity was also confirmed for their osteogenic and adipogenic differentiation as well as their clonogenic capacity (20). For *in vivo* studies, Ad-MSC at passage 3 to 5 were harvested, counted and resuspended in cooled PBS for injection.

### Animal model

We employed three-month old New Zealand White male rabbits (2.5-3.0 kg body weight, Granja San Bernardo, Spain) that were housed in individual cages (0.50 m height, 0.6 m^2^ floor space) and exposed to a 12-hour light/dark cycle. After 2 weeks of adaptation to our facilities, an acute gout flare was induced in 24 anesthetized rabbits by intraarticular injections of 50 mg MSU crystals resuspended in 1ml PBS into each knee, as previously described (11, 22). Immediately after MSU injection, right femoral artery was dissected and cannulated with a 24G gauge needle (Abbocath, Venisystems, Spain). One hour after MSU injection, 12 of these rabbits received a single dose of 2x10^6^ Ad-MSC/Kg, resuspended in 2ml of cooled PBS through the right femoral artery (MSU+MSC group), while the other 12 rabbits received PBS through the same procedure (MSU group). We simultaneously followed 4 sex- and age-matched rabbits, which received intraarticular injections of 1ml PBS in both knees and were employed as controls. All procedures were performed under aseptic conditions and general anesthesia (11, 23). One MSU+MSC rabbit died during the surgical procedure.

Four rabbits from each group (MSU and MSU+MSC groups) were euthanized 24 hours after MSU injections by intracardiac injection of pentobarbital (50 mg/kg, Braun Medical SA, Spain), and were employed for the differential cell count study. Synovial fluid (SF) from these animals was collected after knee joint lavage with 1 ml of cold PBS injected into each joint cavity. All the remaining animals were euthanized 72 h after MSU administration. Then, the SF, and synovial membranes (SM) were collected, and processed for further studies. A piece of SM was fixed in 4% buffered formalin (Sigma) for 24h and then embedded in paraffin. Another SM piece from each knee was immediately frozen in liquid nitrogen (24).

Knee joint swelling was measured using a standardized flexible measuring tape at different time points: just before intraarticular injection and 6, 18, 24, 48 and 72 h after MSU administration. Blood samples were collected from the auricular artery 24 and 72 h after MSU administration.

All the experiments were performed in accordance with the Animal Research Reporting of *In vivo* Experiments (ARRIVE) guidelines and with the National regulation and the Guidelines for the Care and Use of Laboratory Animals, drawn up by the National Institutes of Health (Bethesda, MS, USA) (25). These procedures were approved by the Institutional Ethics and Animal Welfare Committee of IIS-FJD.

### Synovial fluid cell count

Total leukocyte number was calculated in each SF sample staining with Türk’s solution (Sigma-Aldrich, St Louis, Missouri, USA) and counting by LUNA-II™ Automated Cell Counter (Logos Biosystems, Gyeonggi-do, South Korea). Haemorrhagic SF samples were discarded (1 from MSU group, and 2 from MSU+MSC group). Leukocyte differential count was performed in the SF from each knee joint cavity. SF smears were fixed in methanol and subsequently stained with May-Grünwald Giemsa (Sigma-Aldrich, St Louis, Missouri, USA) staining. Ten different pictures in each sample were obtained with a Leica DM 6000 LED instrument (Leica, Microsystems, Inc. Buffalo Grove, IL, USA) in order to calculate the percentage of polymorphonuclear cells (PMNC) and mononuclear cells (MNC) present in each sample.

### Serum C-Reactive Protein (CRP) and synovial PGE_2_ measurement

Serum CRP levels at 24 and 72h after MSU administration were determined with a commercial ELISA kit (ab157726, Abcam, Cambridge, UK) (26). In addition, PGE2 was measured in the synovial tissue. Rabbits’ synovium was ice-cold pulverized (100 mg) and 500 µL 0.1 M phosphate PBS, pH 7.4, 1mM EDTA was added. After centrifugation, PGE_2_ concentration was measured employing ELISA kit (514010, Cayman Chemical, USA, MI).

### Histological evaluation.

SM inflammation was evaluated in hematoxylin-eosin-stained sections by three blinded observers, according to the Krenn score (11, 27). Briefly, lining hyperplasia, tissue cell infiltration and stromal activation were independently evaluated using a subscale graded from 0 to 3 points. The total synovitis score was calculated summing partial grades, with a maximum of 9 points.

### Immunohistochemistry

Vascularization in the SM was evaluated using a monoclonal anti-CD31 antibody (Abcam, Cambridge, UK; clone JC/70A; 1/20 dilution). SM macrophages were stained employing a monoclonal anti-rabbit macrophage RAM11 antibody (Dako, Glostrup, Denmark; 1/100 dilution) and the monoclonal anti-human CD163 antibody (Serotec, Raleigh, NC, USA; clone EDhu-1, 1/500 dilution), as described (26, 28). Arginase-1 presence was examined using a goat polyclonal anti-Liver Arginase (Abcam, Cambridge, UK; 1/500 dilution) antibody (29). NLRP3 was studied in SM macrophages by using a mouse IgG2b anti-human (Adipogen, San Diego, Ca, USA; 1/100 dilution). In brief, 3μm paraffin sections were rehydrated and incubated with blocking solution (PBS 6% sheep serum, 4% BSA). Sections were incubated with the primary antibodies in blocking solution, overnight at 4°C. A biotinylated goat anti-mouse IgG (Amersham, Arlington Heights, IL, USA; 1/800 dilution) was employed as secondary antibody, which was visualized employing ABComplex (Dako, Camarillo, CA, USA). Tissue sections were counterstained with haematoxylin and mounted in DPX medium (VWR International, Leuven, Belgium). For CD31^+^ and Arginase-1^+^ analysis, 10 microphotographs were randomly taken in each tissue sample (20X magnification), while 5 random pictures along lining area were obtained at 40X magnification for macrophage analysis, using a Leica DM 6000 LED instrument (Leica, Microsystems, Inc. Buffalo Grove, IL, USA). Each image was analysed using the Color Deconvolution plugin of ImageJ software (NIH, Bethesda, MD, USA) to calculate the percentage of positive staining relative to the total tissue area. The means of positive area corresponding to each sample was then calculated for each group (24, 30).

### Western-blotting.

Total proteins were extracted from SM, resolved on SDS-PAGE gels and transferred to nitrocellulose membranes in a semi-dry Trans-Blot device (Bio-Rad, Madrid, Spain) as described (24, 28). The following primary antibodies were applied overnight at 4°C: anti-human COX-2 (Santa Cruz Biotechnology, Dallas TX, USA), anti-rabbit IL-6, anti-rabbit TNF, anti-rabbit IL-10, anti-rabbit TGF-β (all from Cloud-Clone Corp; 1/250 dilution); anti-human NLRP3 (AdipoGen, Liestal, Switzerland; 1/1000 dilution); anti-human Caspase-1 (Thermo Fisher Scientific, IL, USA; 1/500 dilution), anti-rabbit IL-18 and IL-1β antibodies (Cloud-Clone Corp, Houston TX, USA; 1/250 dilution). Protein loading control was performed employing EZBlue gel staining reagent (Sigma-Aldrich (11, 24).

### NF-κB activation assay

NF-κB activation was assessed using an ELISA-based TransAM NF-κB p65 kit (Active Motif, CA, USA) in accordance with manufacturer’s protocol. Briefly, nuclear proteins were isolated from total protein extracts and incubated with NF-κB consensus sequences. Then, hybridization was detected by a colorimetric reaction, and quantified by absorbance measurement (31).

### *In vitro* experiments

THP-1 monocyte cells (passage 6 to 11) (American Type Culture Collection, Manassas, Virginia, USA) were grown at 37°C and 5% CO_2_ in RPMI 1640 (Gibco BRL, Grand Island, NY) supplemented with 10% heat-inactivated FBS, 50 units/ml penicillin-streptomycin and 2 mM L-Glutamine (Gibco BRL). THP-1 monocytes were differentiated to macrophages in the presence of 0.5 µM Phorbol 12-myristate 13-acetate (PMA, Sigma-Aldrich) for 3 hours. 1x10^6^ THP-1 cells were seeded in 6 well plates and were incubated O/N at 37°C and 5% CO_2_ in RPMI 1640, 2% heat-inactivated FBS, 50 units/ml penicillin-streptomycin and 2 mM L-Glutamine. 2.5x10^5^ Ad-MSC (passage 3-4) were seeded and incubated into polycarbonate Transwell inserts of 24 mm and 0.4 µm membrane pore size (Corning, New York, USA) O/N in RPMI 1640, 2% heat-inactivated FBS, 50 units/ml penicillin-streptomycin and 2 mM L-Glutamine. For Transwell experiments, 24 h before being used in experiments.

PMA-differentiated THP-1 macrophages were stimulated with 300 µg MSU crystals (Invivogen, San Diego, USA) or vehicle (PBS). One hour after the addition of the crystals, Transwell inserts were placed over THP-1 macrophages in a ratio 1:5 (Ad-MSC: THP1). Cells were co-cultured for 6, 12 or 24 h after the addition of the stimuli.

Human peripheral blood mononuclear cells (PBMCs) were isolated by Ficoll gradient from healthy volunteers. After differentiation employing MCSF 50ng/mL for 6 days, 1x10^6^ cells were seeded on p6 in RPMI 1640 10% FBS. After 24 h, macrophages were stimulated during 4 hours with LPS 1ng/mL, and then 200 µg of MSU crystals were added. One hour after the addition of the crystals, transwell inserts containing MSCs were placed over primary macrophages in a ratio 1:5 (Ad-MSC: PBMC derived macrophage). Cells were cocultured for 24 h after the addition of the stimuli.

### RNA isolation and RT-PCR

RNA was separately isolated from each cell type using TRIzol reagent (Roche Diagnostics, Barcelona, Spain), solubilized in nuclease-free water and quantified with a NanoDrop ND1000 spectrophotometer (Thermo Fisher Scientific, Waltham, Massachusetts, USA). A High-Capacity cDNA Reverse Transcription Kit (Applied Biosystems, San Francisco, California, USA) was used to reverse-transcript 1 µg RNA following manufacturer’s instructions. Step One Plus Detection system (Applied Biosystems, Foster City, CA) was employed to analyze RNA expression by single-reporter real-time PCR. Specific commercial TaqMan^®^ probes were purchased from Applied Biosystems to assess the expression of human IL-1β, TNF, COX-2, PTGES, TGF-β, IL10, IDO and TSG6. RNA expression levels were quantified using the ΔΔCt method, and hypoxanthine-guanine phosphoribosyl-transferase (HPRT) expression as endogenous control.

### Cell culture supernatant ELISA

Supernatants were collected from cocultures between Ad-MSCs and PBMC-derived macrophages. They were centrifuged at 4° for 10 min at 2000 g to remove MSU crystals. Supernatants were analyzed according to the manufacturer’s instructions for PGE_2_ (514010, Cayman Chemical, USA, MI) and IL-10 (ADI-901-036, ENZO, USA, NY).

### Statistical analysis

GraphPad Prism package (8.0 for Windows) was used for statistical analysis. To compare the evolution of joint perimeter between the different groups, two-way analysis of ANOVA variance was employed followed by Bonferroni’s correction. For all other statistical evaluations, an overall Kruskal-Wallis test was undertaken prior to individual Mann-Whitney tests adopting a Bonferroni’s correction. Quantitative data were expressed as mean ± SEM. P values less than 0.05 were considered significant. Our population size calculations (G-power 3.1) gave us a sample size of 4 rabbits per group to have 95% power with p=0.05, according to previous data on SM global histopathological score. Besides SM histopathological score, additional molecular studies were designed, with higher heterogeneity intra-group in the response to MSU, so we decided to increase this number to 8 in MSU-Vehicle and MSU+MSC groups.

## Results

### Effect of intraarterial administration of Ad-MSC in joint and systemic inflammation

**Figure 1:**
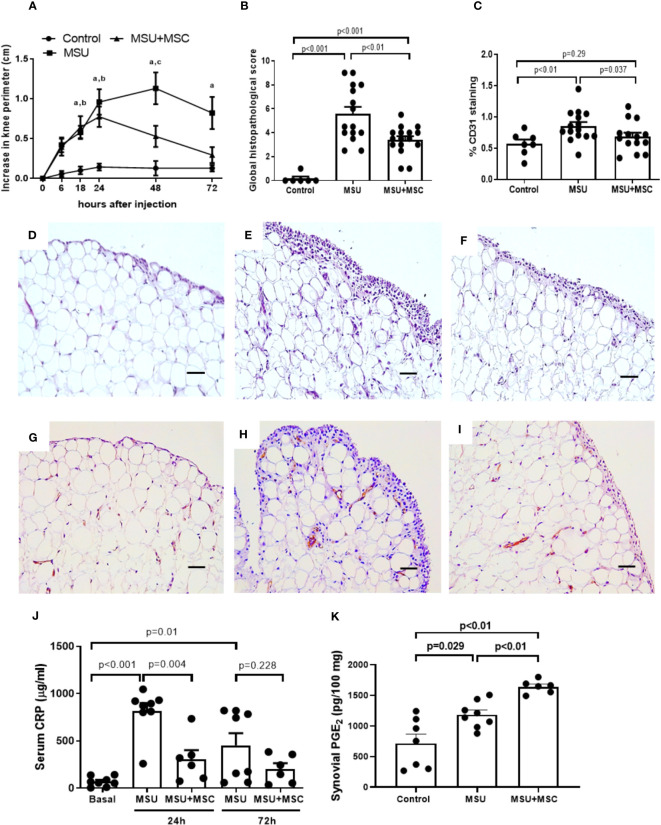
Systemic administration of Ad-MSC through the right femoral artery attenuates synovial inflammation and reduce systemic inflammation in rabbit knees injected with MSU crystals. **(A)** Evolution of joint swelling by increase in knee perimeter in each group of animals along the study. **(B)** Global histopathological score (Krenn Score). **(C)** Analysis of CD31 positive staining in the synovial membrane of each group of animals. N=8-16 joints per group. **(D–F)** Representative sections hematoxylin-eosin staining of the synovium 72 hours after intraarticular injections, from: D, Control; E, MSU; and F, MSU+MSC groups. **(G–I)** Representative sections of immunohistochemical staining of CD31 in the synovium 72 hours after intraarticular injections, from: G, Control; H, MSU; and I, MSU+MSC groups. Scale bars=50μm. **(J)** Serum C-Reactive protein concentration levels at 24h and 72h after MSU intraarticular injections. N= 8 animals per group **(K)** Synovial membrane PGE_2_ concentration levels at 24h after MSU intraarticular injection. N= 4 animals per group. Bars show the mean and SEM. *p<0.05 vs. Control, #p<0.05 vs. MSU. MSC: mesenchymal stem cells; MSU: monosodium urate.

We first analyzed whether Ad-MSC administration has any effect on joint swelling and SM inflammation in MSU crystal-induced arthritic rabbits. Our data showed that MSU induced a marked increase in knee perimeter in a few hours, reaching a 1.1 cm peak 48 h after injections (**Figure 1A**). In the MSU group, SM histological alteration was evident 72 h after MSU injection (**Figure 1B**), characterized by hyperplasia of lining layer, stromal activation with increased cellularity, irregular adipocytes and increased fibrotic component in the synovial stroma, and a boost of infiltrating cells, in comparison to control SM (**Figures 1D, E**). We then specifically assessed synovial neo-angiogenesis by the analysis of %CD31^+^ cells. In line with the synovitis score, we observed an increased presence of newly formed vessels in the SM of MSU rabbits, in comparison to controls (**Figures 1C, G, H**). Furthermore, intraarticular MSU injection increased serum CRP concentration at 24 h, a measurement of systemic inflammation, while at 72 h significantly dropped (**Figure 1J**). A single dose of Ad-MSC evoked a significant decrease in knee swelling intensity at 48 h, and a diminution in the duration of the flare, since baseline values were reached 72 h after MSU injections, in comparison to untreated knees (**Figure 1A**). A clear decrease in the histopathological lesions in the SM was also observed, with amelioration of synovial hypertrophy and in the accumulation of inflammatory cells (**Figures 1B, E, F**), while a significant reduction of vascularization was induced by Ad-MSC treatment (**Figures 1C, H, I**). Furthermore, Ad-MSC had an appreciable effect on the systemic inflammation evoked 24 h after MSU injection, and a similar but not statistically significant trend was observed 72 h post injury (**Figure 1J**).

In order to study the early effect of Ad-MSC administration in PGE_2_, which has been implicated in the anti-inflammatory effect of MSC, we measured PGE_2_ concentration in the synovium 24 hours after MSU injection. We observed a significant increase in synovial PGE_2_ levels in the MSU group (**Figure 1K**). Notably, treatment with Ad-MSCs resulted in a significant super-induction in PGE_2_ concentration in the synovium in comparison to MSU group (**Figure 1K**).

### Effect of intraarterial administration of Ad-MSC on leukocyte population in the synovial fluid of rabbit knees

**Figure 2:**
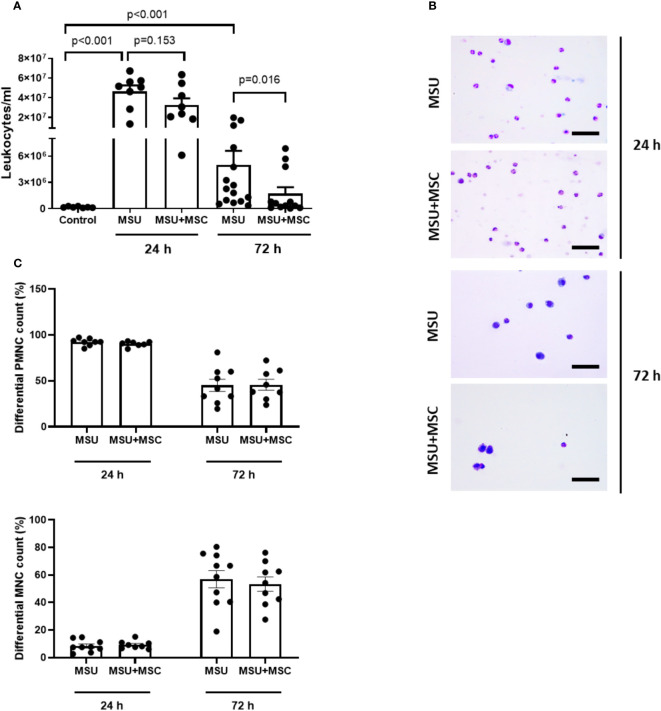
Systemic administration of Ad-MSC reduces total leukocyte population at 72 h but do not alter percentage of PMN-MNC cells. **(A)** Total leukocyte count in synovial fluid at 24 h and 72 h. **(B)** Representative images of synovial fluid samples of MSU and MSU+MSC groups stained with May-Grünwald Giemsa at 24 h and 72 h. **(C)** Distribution of PMNC and MNC at 24 h and 72 h of MSU and MSU+MSC groups. Scale bar = 50 µm. MSC: mesenchymal stem cells; MSU: monosodium urate; PMNC: polymorphonuclear cell; MNC: mononuclear cell.

We performed a sequential study of leukocyte infiltrate in the SF of each rabbit knee at 24 and 72 h after MSU injections. The total number of leukocytes dramatically increased in the SF of MSU injected rabbits 24 h after injury (**Figure 2A**), while no significant differences were found in the number of leukocytes between MSU and MSU+MSC groups at this time point. After 72 hours, the number of leukocytes in the SF of the MSU group remained significantly elevated compared to the control group, while a significant decrease was observed in the MSU-MSC group in comparison to the MSU at the same time point (**Figure 2A**). Differential cell count revealed a significant change in the type of infiltrating cells along the study (**Figure 2B**). As can be observed, 24 h after MSU injection SF infiltrate was mainly composed of PMNC, comprising almost 90% of total leukocytes present in both MSU and MSU-+MSC knees, while around 7% were MNC. However, 72 h after injury, the percentage of PMNC dropped under the 50% evenly in both groups, and the presence of MNC was around 55% (**Figures 2B, C**), with a similar distribution between these groups.

### Effect of Ad-MSC treatment in the presence of infiltrated M1 and M2 macrophages in the synovial membrane of arthritic rabbits

**Figure 3:**
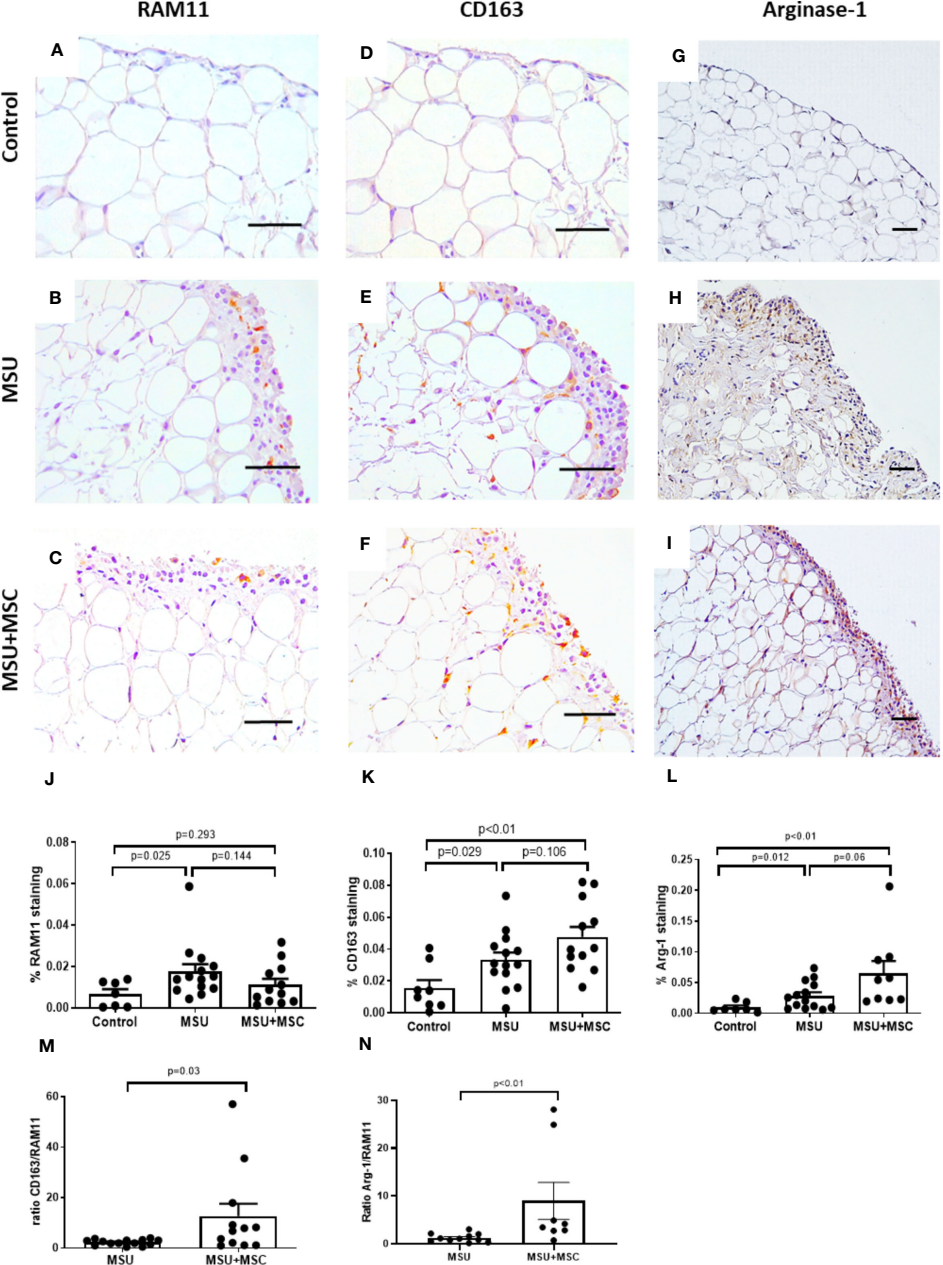
Immunohistochemical analysis of macrophage population within synovial membranes at 72h after MSU injection. **(A–C)** Representative images of RAM11 antigen staining in the synovium of Control **(A)**, MSU **(B)** and MSU+MSC **(C)** groups. **(D–F)**, Representative images of CD-163 antigen staining in the synovium of Control **(D)**, MSU **(E)** and MSU+MSC **(F)** groups. **(G–I)**, Representative images of arginase-1 staining in the synovium of Control **(G)**, MSU **(H)** and MSU+MSC **(I)**. Scale bar=50μm. **(J)** Densitometric analysis of RAM11 staining percentage, and **(K)** CD163 staining percentage in the SM of each group of animals. **(L)** Percentage of arginase-1 staining in lining layer of each group of animals. **(M)** Ratio of CD163 to RAM11 positive staining. **(N)** Ratio of Arginase-1 to RAM11 positive staining. Bars show the mean and SEM. MSC, mesenchymal stem cells; MSU, monosodium urate.

As gouty arthritis is characterized by extensive macrophage infiltration, we analyzed the presence of macrophages in different phenotypes in the rabbit SM employing RAM11 antibody, that recognizes rabbit macrophages in all different phenotypes; and anti-CD-163, a monocyte and macrophage marker which has been reported to be overexpressed in M2a macrophages (32, 33). RAM 11 positive cells were mainly located in the SM lining layer of MSU rabbits, while some positive cells were observed surrounding adipocytes forming “crown-like” structures close to the sublining vessels. Thus, macrophage presence was quantified in the SM lining layer. RAM11 positive staining was significantly incremented in MSU rabbits when compared to controls (**Figures 3A, B, J**), although it was not significantly different to that found in MSU-MSC rabbits, neither compared to the MSU group, nor compared to control animals (**Figures 3C, J**). We also examined CD163 along the synovial lining layer, where macrophage staining was relevant. In this case, both MSU and MSU+MSC rabbits showed an increased presence of CD163 positive cells, in comparison to control group (**Figures 3D–F, K**). However, no statistically significant differences were observed between the arthritic groups (**Figure 3K**). To assess the pattern of polarization to M2 cells in the synovial macrophages of the arthritic animals, we analyzed the ratio CD163/RAM11 staining. **Figure 3M** shows a relevant increased ratio in the MSU+MSC group compared to MSU, indicating a major presence of M2a macrophages in the Ad-MSC treated animals (34). Additionally, we analyzed the presence of arginase-1 in the SM, which has been considered a M2 marker in rabbit (35). We observed that arthritic animals overexpressed this protein along the lining layer of SM (**Figures 3G–I, L**). Our results indicate that Ad-MSC treated animals showed a clear trend to an increased expression of arginase-1 compared to untreated MSU group. Regarding the ratio arginase-1/RAM11 staining, we observed an augmented ratio in MSU+MSC group in comparison with MSU. (**Figure 3N**).

### Modulation of the synthesis of different pro- and anti-inflammatory mediators by Ad-MSC in the SM of arthritic rabbits

**Figure 4:**
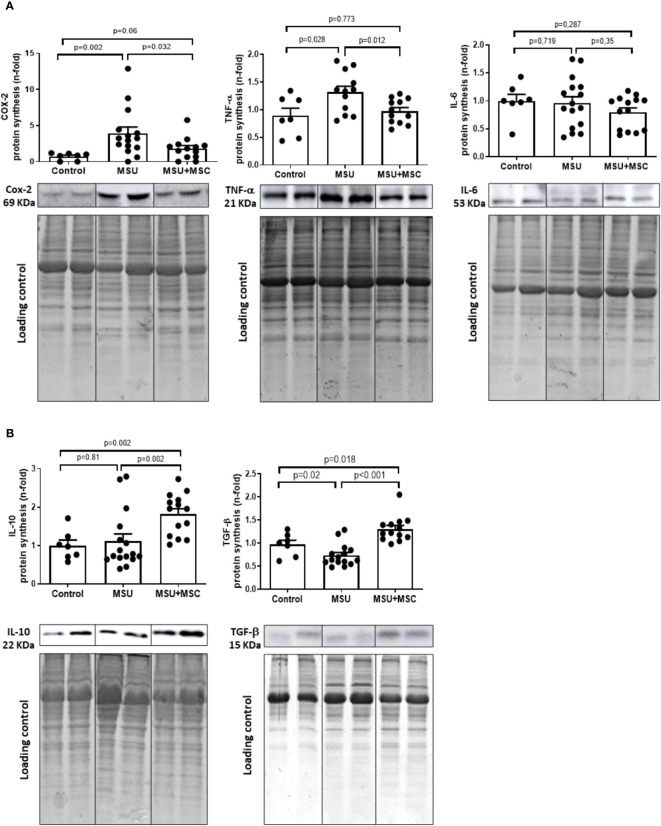
Ad-MSC modulates pro- and anti-inflammatory cytokine profile in synovial membranes of arthritic rabbits. Representative western blot of pro-inflammatory cytokines **(A)** COX-2, TNF, IL-6 and M2 anti-inflammatory cytokines levels **(B)** IL-10 and TGF-β. EZ blue staining was used as protein loading control and to normalize the results, which are expressed as a fold-change of the Control group. Bars show the mean and SEM. COX-2, Ciclooxygenase-2; IL, interleukin; TGF-β, tumor growth factor-β, TNF-α, tumor necrosis factor α; MSC, mesenchymal stem cells; MSU, monosodium urate.

As expected, MSU rabbits showed a marked increase in the synthesis of pro-inflammatory cytokines in the SM at the time of sacrifice. COX-2 and TNF presence were increased in these rabbits in comparison to control ones (**Figure 4A**), while no differences were observed in the presence of IL-6 levels between the groups studied. Meaningfully, the administration of Ad-MSC induced a statistically significant decrease in the presence of COX-2 and TNF in the SM of arthritic rabbits, in comparison to untreated animals. In addition, IL-10 and TGF-β, strongly related to M2 polarization and associated to the inhibition of the inflammatory process and tissue regeneration, were significantly incremented in the SM of the MSU+MSC group 72 h after the intraarticular injection, in comparison to both MSU and control groups (**Figure 4B**).

### Protein expression of NLRP3 inflammasome pathway in the SM of arthritic rabbits after Ad-MSC infusion

**Figure 5:**
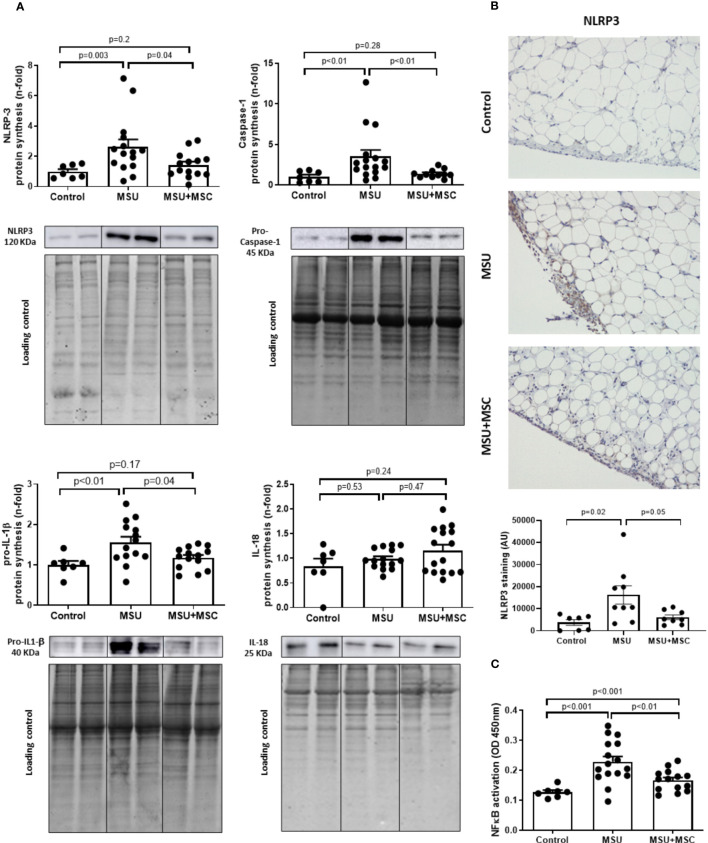
Regulation of inflammasome components and NF-kB pathway by Ad-MSC in the synovium of MSU-injected rabbits. **(A)** Protein expression of inflammasome components is modulated in arthritic rabbits treated with Ad-MSC. Activated NLRP3, pro-caspase-1, IL-1β, and IL-18 were assessed by western blot. EZ blue staining was used as protein loading control. Results are normalized by EZ blue staining and expressed as a fold-change of the healthy group. Bars show the mean and SEM. **(B)** Representative images and quantification of NLRP3 antigen staining in the synovium of Control, MSU and MSU+MSC groups. Bars show the mean and SEM **(C)** NF-kB p65 was analyzed by TransAM kit assay. Results are expressed in optical density (OD) units. Bars show the mean and SEM. IL, interleukin; NLRP3, NLR Family Pyrin Domain Containing 3; MSC, mesenchymal stem cells; MSU, monosodium urate.

Since it has been demonstrated that MSU deposit induces the inflammatory flare in the SM through the activation of the NLRP3 inflammasome (7, 8), we determined whether Ad-MSC administration was able to decrease inflammasome pathway in gouty arthritic rabbits. Our results indicate that NLRP3, pro-Caspase-1 and pro-IL-1β protein synthesis were significantly induced in the SM of MSU rabbits, while no differences were observed in the level of IL-18, in comparison to control animals (**Figure 5A**). The administration of Ad-MSC induced a clear decrease in the presence of these mediators, in comparison to untreated MSU group (**Figure 5A**). It is relevant that positive NLRP3 labelling is observed in lining and sublining macrophages, and in macrophages that appear forming Crown-like pro-phagocytic structures around adipocytes. A higher signal intensity for NLRP3 is confirmed in the MSU group, whereas it is significantly decreased after MSC treatment. (**Figure 5B**).

In addition, we tested whether the activation of NF-κB, the downstream nuclear factor of the inflammasome pathway, was regulated by this treatment. According to our data, MSU administration increased the synovial activation of NF-κB, while a significant inhibition was observed in the MSU-MSC group in comparison to MSU animals (**Figure 5C**).

### Effect of Ad-MSC on THP-1 derived macrophages stimulated with MSU crystals

**Figure 6:**
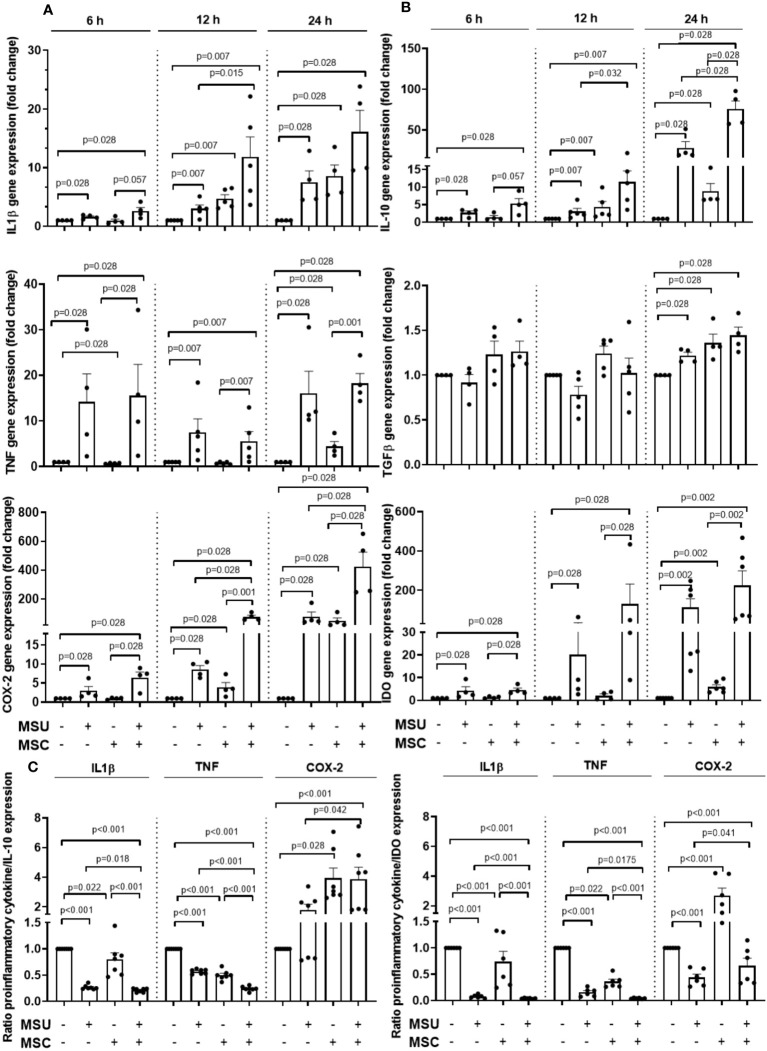
Ad-MSCs modulate MSU crystals-stimulated THP-1 cells inflammatory activity *in vitro*. Gene expression levels of different pro- **(A)** and anti- **(B)** inflammatory cytokines produced by THP-1 derived macrophages at 6, 12 and 24 h after MSU stimuli with Ad-MSC transwell co-culture system. **(C)** Ratio of pro-inflammatory cytokines (IL-1β, TNF, COX-2) to anti-inflammatory cytokines (IL-10 and IDO). Bars show the mean and SEM (n=4-5 independent experiments). MSC, mesenchymal stem cells; COX-2, Ciclooxygenase-2; IL, interleukin; NLRP3, NLR Family Pyrin Domain Containing 3; TGF-β, tumor growth factor-β, TNF-α, tumor necrosis factor α; MSU, monosodium urate.

Since macrophage characteristics seemed to be modulated by Ad-MSC administration *in vivo*, we next investigated whether Ad-MSC were able to modulate the inflammatory status of human macrophages stimulated by MSU crystals using a transwell co-culture system. Firstly, THP-1 monocytes were PMA-differentiated into macrophages one day before the addition of the MSU crystals or its vehicle, and the gene expression of different cytokines and inflammatory mediators was analyzed after 6, 12 and 24 hours in THP-1 macrophages after the addition of MSU. As can be observed in **Figures 6A and B**, MSU presence induced a time-dependent increase in the expression of the pro-inflammatory mediators IL-1β, TNF-α and COX-2. Besides, an increase in the presence of the anti-inflammatory markers IL-10 and IDO was also induced by MSU at all the time points studied, in comparison to unstimulated macrophages, while TGF-β was significantly induced after 24 hours of stimulation with MSU. Ad-MSC inserts were transferred over THP-1 macrophages hour after MSU crystals or vehicle stimulation, trying to mimic the *in vivo* setting. Interestingly, just the presence of Ad-MSC increased the mRNA expression of IL-1β and COX-2 in the vehicle-treated macrophages in a time-dependent manner in a similar amount to that observed for MSU stimulation, in comparison to the expression observed for unstimulated macrophages (**Figures 6A, B**). The presence of Ad-MSC over MSU-stimulated macrophages further increased the gene expression of IL-1β and COX-2, in comparison with MSU-stimulated macrophages, at the different times studied. Regarding TNF, the addition of Ad-MSC did not significantly modify its gene expression in the time-points studied. The gene expression of the anti-inflammatory mediators IL-10 and IDO was also further up-regulated by Ad-MSC in comparison with the induction observed with MSU, while TGF-β expression was similar to that observed in MSU-stimulated macrophages (**Figures 6A, B**). To establish what could be the net effect of the co-incubation of Ad-MSC and macrophages in this pro-inflammatory milieu, we studied the ratio of the expression level of pro-inflammatory cytokines to either IL-10 or IDO in the macrophages. While a pro-inflammatory net effect was observed at short time points (6 hours), we observed that the ratios IL-1β/IL-10 and TNF/IL-10 were dramatically reduced in MSU-stimulated macrophages in the presence of Ad-MSC after 24 hours, in comparison to MSU-stimulated cells (**Figure 6C**). In the same line, the ratios IL-1β/IDO and TNF/IDO in MSU-stimulated cell in co-culture at 24 hours were further decreased in comparison to those observed for MSU-stimulated macrophages in the absence of Ad-MSC (**Figure 6C**).

### Effect of Ad-MSC effect on PBMC-derived macrophages stimulated with MSU crystals

**Figure 7:**
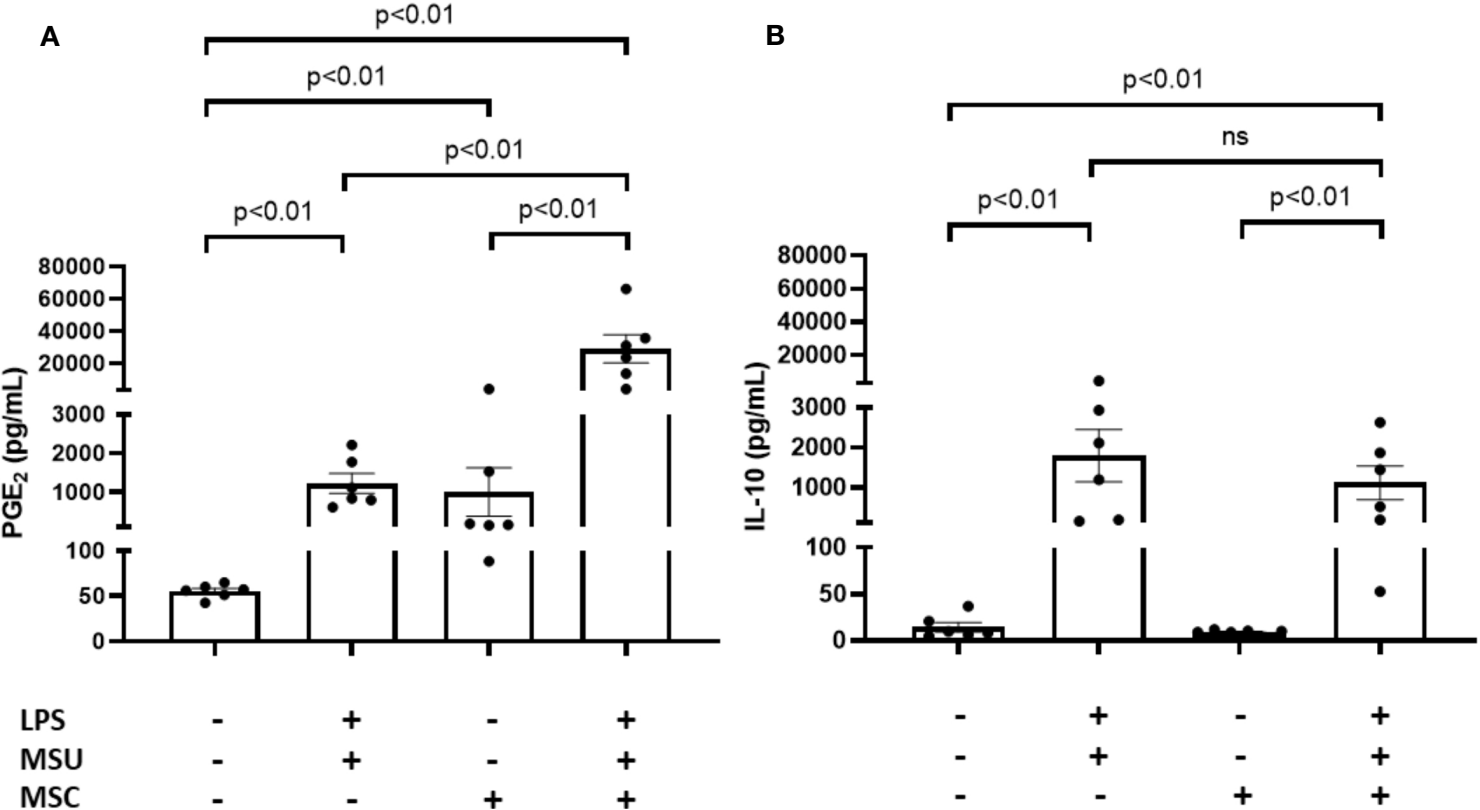
Ad-MSCs modulate PGE_2_ release in MSU crystals-stimulated PBMC derived macrophages *in vitro*. Quantification of PGE_2_ **(A)** and IL-10 **(B)** levels in cell supernatant from coculture system between Ad-MSCs and PBMC derived macrophages after 24 h MSU stimulation. Bars show the mean and SEM (n=6). MSC, mesenchymal stem cells; MSU, monosodium urate; LPS, lipopolysaccharide.

In order to confirm the ability of Ad-MSCs to super-induce both a proinflammatory and an anti-inflammatory response in primary macrophages, we measured PGE_2_ and IL-10 release to culture media in transwell cocultures employing human PBMC-derived macrophages. As can be observed in **Figure 7A**, LPS+MSU-stimulated macrophages induced a significant release of PGE_2_ to culture media after 24 h of coculture, in comparison to unstimulated cells. The presence of Ad-MSCs significantly super-induced PGE_2_ secretion in comparison to LPS+MSU stimulated cells (**Figure 7A**). Regarding IL-10, a similar increase in the release of this anti-inflammatory cytokine was observed in stimulated-primary macrophages in the presence or absence of MSC in comparison to unstimulated macrophages (**Figure 7B**).

### Effect of MSU stimulation on Ad-MSC co-cultured with THP-1 derived macrophages

**Figure 8:**
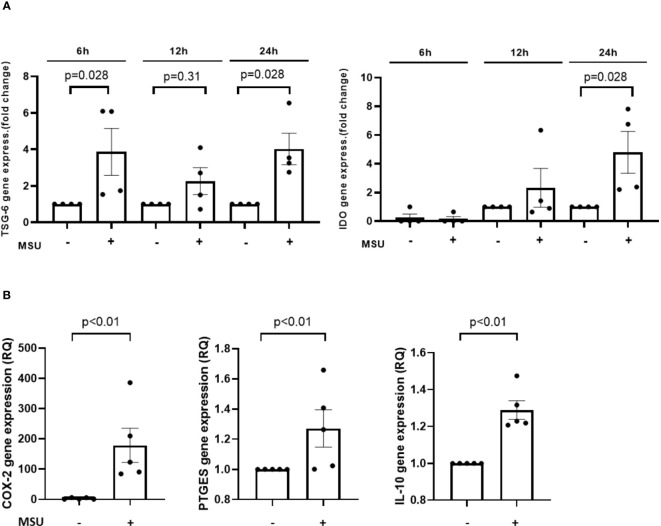
Transwell Ad-MSC express immunosuppressive and proresolutive genes after MSU-stimulated THP-1 macrophage *in vitro*. Gene expression levels of TSG-6, IDO at 6, 12 and 24 h **(A)** and COX-2, PTGES and IL-10 at 24 h **(B)** corresponding to Ad-MSC in presence of THP-1 derived macrophages after stimuli in transwell co-culture system. Bars show the mean and SEM (n=4-5). MSC, mesenchymal stem cells; MSU, monosodium urate.

Finally, we investigated the Ad-MSC-derived factors that could potentiate the anti-inflammatory response in macrophages. As can be observed in **Figures 8A, B**, the presence of MSU induced TSG-6, IDO, COX-2, PTGES and IL-10 gene expression in Ad-MSC that were co-cultured with THP-1 derived macrophages after 24 hours of stimulation, in comparison to vehicle-stimulated cells.

## Discussion

The results of many clinical trials employing stem cell-based treatments in chronic inflammatory and autoimmune diseases are variable and inconsistent with the postulated benefits (36–39). This reveals a greater need for testing these treatments in robust preclinical models that can better predict the clinical translation of these therapies.

In chronic conditions, macrophages are maintained in a delicate back and forth transition between pro-inflammatory M1 and pro-resolutive M2 phenotypes (38, 40). The complex integration of both the adaptive and the innate immune responses, coexisting in a continuum induction and resolution processes, makes difficult to clearly dissect the mechanisms responsible for the improvement in the progression of the chronic conditions, or to predict the response to MSC administration.

Our data demonstrate that systemic administration of Ad-MSC improve joint inflammation in an acute model of gout induced by intra-articular injection of MSU. Ad-MSC significantly decreased joint swelling at the peak time of knee inflammation. After 72 hours, the inflammatory flare was completely resolved in treated animals, while a marked articular inflammation was still present in untreated ones. The histopathological lesions of the SM were also significantly prevented by this treatment, as it was the induction of synovial neo-angiogenesis.

Although scarce data exist on the effect of MSCs in acute models of sterile inflammation in joint diseases, previous data suggest that innate activation in the myeloid compartment may be the first target in MSC-based therapy in experimental colitis (41). Ad-MSC administration decreased disease activity in dextran sulphate sodium-induced colitis, even in animals lacking mature B and T cells (41). It suggests that myeloid cells could be specifically targeted in MSC therapy. In our innate-triggered inflammatory experimental model, that entirely reproduce human clinical process (11, 42, 43), synovial inflammation is solely induced by the presence of urate crystals, whose ingestion by mononuclear phagocytes leads to inflammasome activation (43). According to our data, the anti-inflammatory effect of MSCs is based on an acceleration of resolution process, which is often directed by phenotypic switching towards pro-resolving and anti-inflammatory macrophages. In fact, *in vitro* studies have suggested that direct polarization to M2 macrophages could be the mechanism by which MSCs could have a beneficial effect in experimental chronic arthritis (19). The measurement of specific markers of M2 anti-inflammatory macrophages showed that the presence of CD163 and Arg-1 was increased in the SM of MSU animals, as can be conceivable in a self-limited disease. Interestingly, MSC treatment exacerbated the presence of these markers in the SM of the animals at 72 h. MSC administration induced a clear decrease in the pro-inflammatory markers COX-2 and TNF in the SM. Simultaneously, a significant induction in the synthesis of M2 polarization markers IL-10 and TGFβ was observed, which has been associated with the shutdown of the gouty inflammation process (44–46). Both IL-10 and TGFβ have been described as M2 macrophage markers in rabbits (35), associated with the anti-inflammatory and pro-resolutive profile of these cells (47). MSCs have been reported to increase M2 macrophage polarization *in vivo* and to decrease the presence of inflammatory mediators after acute tissue injury in experimental models of myocardial infarction, stroke or sepsis-induce lung damage (48–50). Therefore, this is the first study demonstrating that MSC administration induce *in vivo* M2 macrophage polarization in the arthritic synovium, suggesting that this can be the mechanisms by which MSC shortens and decreases the intensity of the inflammatory acute flare induced by MSU.

Regarding the inflammatory cell count in the synovial fluid, we observed that the percentage of the cell types involved in the gout flare, PMNCs and MNCs, at 24 and 72 hours, was similar to that described in patients in the first hours after the flare-up (51). Surprisingly, MSCs had no effect on PMNC influx into the inflammatory exudate at 24 h, contrary as reported in different studies of acute inflammation (52–54). A similar percentage of each cell type was observed after 24 and 72 h in MSU and MSU+MSC groups, suggesting that MSC did not exert a specific intervention on cell-type recruitment.

These results in synovial fluid are in line with the idea that a short-term fuelling of pro-inflammatory and chemotactic factors may generate a more efficient milieu in resolving inflammation (55). Ad-MSC priming with pro-inflammatory stimuli enhanced their immunomodulatory ability (52). Serhan and colleagues proposed that a further increase in PGE_2_ levels in a PMNC-rich inflammatory ambient would facilitate the switch to pathways of active resolution of inflammation (56–58). In this context, Ad-MSC would rapidly increase the release of inflammation-dependent resolution factors, such as PGE_2_, which could trigger a more efficient anti-inflammatory and pro-resolutive response (59). In this sense, we showed that MSCs administration induced an early super-increase in PGE_2_ concentration in the synovium 24 hours after MSU injection. It was followed by an increase in the expression of anti-inflammatory and pro-healing factors, such as IL-10 and TGF-β in the tissue at 72 hours, which was not observed in the untreated animals. These data support the hypothesis that MSC would decrease inflammation through an increase in the release of this lipid mediator in the tissue (60).

To test this hypothesis, we designed *in vitro* experiments co-culturing THP-1 with Ad-MSC. MSU crystals were able to induce an increase in the gene expression of proinflammatory genes, such as IL-1β, TNF and COX-2 in THP-1 derived macrophages since 6 hours of stimulation, in a time-dependent manner. The addition of Ad-MSC co-cultured with MSU-stimulated macrophages increased IL-1β expression in comparison to MSU-stimulated macrophages. Regarding COX-2, a marked super-induction was observed after MSC addition at all the time points studied. An induction of the inflammatory response by MSC in stimulated macrophages has been scarcely described in the literature. Different papers described a decreased expression of TNF or IL-1β induced by these cells (18, 61). In our experiments, MSCs also increased the gene expression of the anti-inflammatory mediators IL-10, TGF-β and IDO in MSU-stimulated macrophages, which have been involved in the resolution of inflammation, M2 polarization, and tissue repair (35, 61). The net effect induced by MSCs on cultured macrophages was significantly anti-inflammatory, decreasing the pro-inflammatory (IL-1β/TNF)/anti-inflammatory (IL10/IDO) ratios. However, this was not the case for COX-2 expression, suggesting that PGE_2_ synthesis was not inhibited but further enhanced by MSC presence in the co-culture system. Previous data have demonstrated that PGE_2_ release by MSC is a key event for its anti-inflammatory and pro-resolutive effect (60, 62, 63). Moreover, our data demonstrates that COX-2 and PTGES expression were highly and rapidly induced in MSC and in MSU-stimulated THP-1 cocultured with MSC, suggesting that both cell types could contribute to a rapid PGE_2_ release to the inflammatory milieu that we observed *in vivo*.

A limitation of this study is the use of a macrophage cell line instead of primary cells, due to the different expression profile of THP-1 markers and secretomes compared to primary macrophages. In order to move towards a more physiological situation, we replicated some results using primary human PBMC-derived macrophage cultures. We showed that co-culture of Ad-MSCs with MSC-stimulated macrophages induced both an anti-inflammatory response and a super-induction of PGE_2_ release, in line with the findings using THP-1-derived macrophages.

All in conjunction, these data suggest that the acceleration on gout flare resolution induced by Ad-MSC in the rabbits after 72 h could be preceded by a further increase in the release of pro-inflammatory mediators such as PGE_2_. From this point of view, our data raise doubts about the use of NSAIDs in patients with acute gout flare.

Ad-MSCs administration decreased the protein presence of different components of NLRP3 inflammasome, such as NLRP3 protein and caspase-1, and inhibited NF-κB activation in the SM of the gouty rabbits. Accordingly, IL-1β levels were decreased in the Ad-MSCs treated rabbits after 72 h of disease induction. The inhibition of NLRP3 inflammasome by MSCs in sterile acute inflammatory disease has been scarcely investigated *in vivo*. *In vitro*, MSCs inhibited NLRP3 activation in LPS stimulated macrophages (60). In experimental peritonitis or acute liver injury during sepsis, MSC administration decreased NLRP3 inflammasome activation and NF-κB signaling in the damaged tissue, probably through the release of PGE_2_ in response to the inflammatory milieu (64–66). Our data show that MSCs decreased NLRP3 inflammasome activation in regions of high macrophage density in the SM of arthritic rabbits.

**Figure 9:**
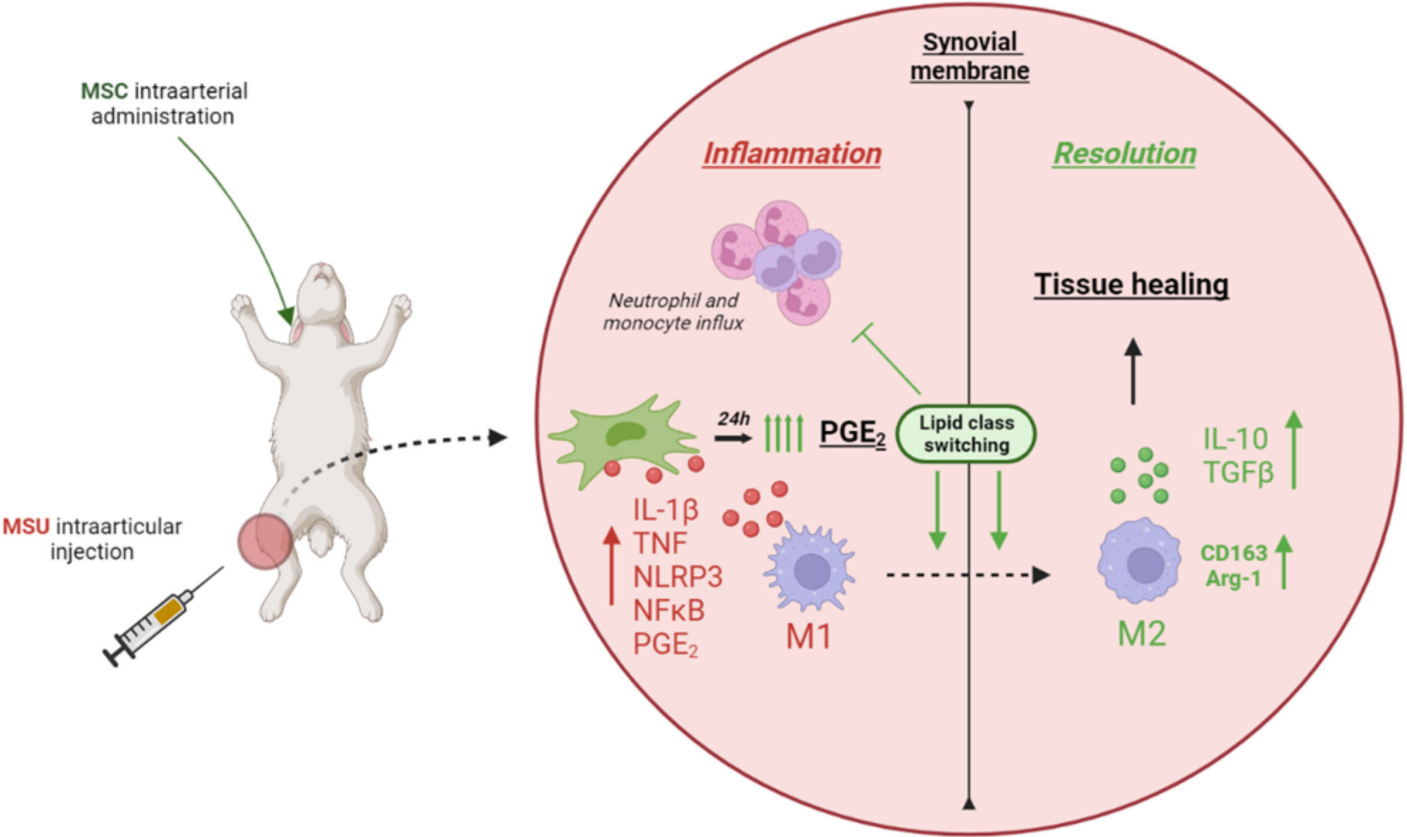
Graphical conclusion.

In conclusion, treatment with a single systemic dose of Ad-MSC decreased the intensity and the duration of the inflammatory response in an acute gouty arthritis model in rabbits through an early marked increase of COX-2 expression and PGE_2_ release. This could be the mechanism by which MSCs suppressed NF-kB activity, inhibited NLRP3 inflammasome, and promoted the presence of M2 alternative macrophages (**Figure 9**).

MSC administration could be a therapeutic opportunity to improve joint outcome after acute flares for patients in whom the possibility of a conventional treatment is very limited, as in polymorbid, elderly patients, and patients with renal pathology or with adverse reaction to NSAIDs. In addition, this work opens new pathways to unravel the mechanisms by which Ad-MSCs accelerate inflammation resolution.

## Data availability statement

The original contributions presented in the study are included in the article/supplementary material. Further inquiries can be directed to the corresponding authors.

## Ethics statement

The animal study was reviewed and approved by Animal Research Reporting of *In vivo* Experiments (ARRIVE) guidelines and with the National regulation and the Guidelines for the Care and Use of Laboratory Animals, drawn up by the National Institutes of Health (Bethesda, MS, USA).

## Author contributions

RL, JPM and IB-A designed the experiments, analyzed and, interpreted the results, and wrote and revised the manuscript. JPM, IB-A and SP-B were primarily responsible for carrying out all experimental procedures and editing and revising of the manuscript. RY, MF-G and DG-O were responsible for MSC obtention and management. RL, GH-B and AM interpreted, revised, and edited the manuscript.
