# Corrigendum: MSC therapy ameliorates experimental gouty arthritis hinting an early COX-2 induction

**DOI:** 10.3389/fimmu.2023.1345777

**Published:** 2023-12-06

**Authors:** Juan Pablo Medina, Ismael Bermejo-Álvarez, Sandra Pérez-Baos, Rosa Yáñez, María Fernández-García, Damián García-Olmo, Aránzazu Mediero, Gabriel Herrero-Beaumont, Raquel Largo

**Affiliations:** ^1^ Bone and Joint Research Unit, Rheumatology Dept, IIS-Fundación Jiménez Díaz Universidad Autonoma de Madrid (UAM), Madrid, Spain; ^2^ Hematopoietic Innovative Therapies Division, Centro de Investigaciones Energéticas, Medioambientales y Tecnológicas (CIEMAT) and Centro de Investigación Biomédica en Red de Enfermedades Raras (CIBER-ER), Madrid, Spain; ^3^ Advanced Therapies Dept, IIS-Fundación Jiménez Díaz UAM, Madrid, Spain; ^4^ New Therapies Laboratory, IIS-Fundación Jiménez Díaz UAM, Madrid, Spain; ^5^ Department of Surgery, Fundación Jiménez Díaz University Hospital, Madrid, Spain; ^6^ Department of Surgery, School of Medicine UAM, Madrid, Spain

**Keywords:** mesenchymal stem cells (MSCs), innate inflammation, macrophage, polarization, inflammasome, prostaglandin E2, COX-2

In the published article, there was an error in the Funding statement. The funding statement was displayed as “This study was supported by research grants from the Instituto de Salud Carlos III (PIE15/00048; PI15/00770; PI18/00261; PI20/00349; PI22/00352), co-funded by Fondo Europeo de Desarrollo Regional (FEDER).” The correct statement is “This study was supported by research grants from the Instituto de Salud Carlos III (PIE15/00048; PI15/00770; PI18/00261; PI20/00349; PI22/00352), co-funded by the European Union.”

The authors apologize for this error and state that this does not change the scientific conclusions of the article in any way. The original article has been updated.

